# The complete mitochondrial genome of *Melanostoma mellinum* (Linnaeus, 1758) (Diptera: Syrphidae) and phylogenetic analysis

**DOI:** 10.1080/23802359.2022.2107452

**Published:** 2022-09-15

**Authors:** Hanyue Liu, Le Zhao, Gang Li, Yicheng He, Keke Huo

**Affiliations:** a School of Biological Science and Engineering, Shaanxi University of Technology, Hanzhong, China; b College of Life Sciences, Shaanxi Normal University, Shaanxi, China

**Keywords:** *Melanostoma mellinum*, Syrphidae, mitogenome, phylogenetic relationship

## Abstract

In this study, the complete mitochondrial genome (mitogenome) of *Melanostoma mellinum* (Linnaeus, 1758) was sequenced using the-next generation sequencing technology. The assembled mitogenome of *M*. *mellinum* has a total length of 16,055bp and contains 13 protein-coding genes (PCGs), 22 transfer RNA genes (tRNAs), and 2 ribosomal RNA genes (rRNAs). The results of phylogenetic reconstruction based on the combined mitochondrial gene dataset indicated that *M. mellinum* belongs to *Melanostoma* genus with a close relationship to *Melanostoma orientale*, but the monophyly of the tribe Bacchini is not well supported.

Syrphidae (Insecta, Diptera) comprise almost 6200 described species worldwide (Evenhuis and Pape 2021) and are well-known by nature enthusiasts and researchers because they provide several crucial ecosystem services (Dunn et al. 2020), such as pollination, biological control of pests and organic matter recycling (Inouye et al. 2015; Moerkens et al. 2021). Among the subfamily Syrphinae, *Melanostoma mellinum* (Linnaeus, 1758) is a small hoverfly with yellow and black markings, which can be well distinguished from other *Melanostoma* species by the following morphological features: face, scutellum, and mesonotum shining black; male adults have nearly square yellow markings on abdominal segments 3 and 4; female adults have inverted yellow triangle markings on abdominal segments 3 and 4 (Huo et al. 2007). The systematic status of the genus *Melanostoma* is still controversial, as this genus has been placed into the subfamily Melanostominae (Williston 1885), tribe Melanostomini (Hull and Riley 1949; Vockeroth 1969, 1990), or classified into the tribe Stenosyrphini (Goffe 1952), or Bacchini (Rotheray and Gilbert 1989). The monophyletic status of the tribe Bacchini is also challenged by the molecular phylogenetic works (Thompson and Skevington 2014; Ståhls et al. 2003). In this study, we obtained the complete mitogenome data of *M. mellinum* and build the phylogenetic tree to reconstruct its evolutionary relationships.

The specimen of *M. mellinum* was collected from the Changqing National Nature Reserve (107°17’E, 33°19′ N) in 2019, and stored in the Museum of Zoology and Botany, Shaanxi University of Technology, Hanzhong, China (SUHC) (Accession number: R81, https://www.snut.edu.cn/, Le Zhao, email: Lezhao@snut.edu.cn). The genomic DNA of *M. mellinum* was extracted using the DNeasy kit (Qiagen, Hilden, Germany), then paired-end libraries (2 × 150 bp) with 400 bp insert sizes were constructed and sequenced by an lllumina HiSeq 4000 platform. We used the software MITOZ (Meng et al., 2019) to assemble and annotate the complete mitogenome, with the putative control region being delineated by tRNA boundaries.

The complete mitogenome of *M. mellinum* was 16,055 bp in length, including 37 typical mitochondrial genes (13 PCGs, 22 tRNAs, 2 rRNAs) and a putative AT-rich control region (D-loop), which has been deposited in GenBank (accession number: OK032510). The base composition of *M. mellinum* mitogenome was 41.2% A, 40% T, 10.6% C, and 8.2% G, with a positive AT-skew (0.014) and a negative GC-skew (−0.123), and all genes were arranged in the same order like other syrphids (Le and Gang, 2020; Zhou et al. 2021). Eight overlaps and 8 intergenic spacers were found in the mitogenome of *M. mellinum*, with the longest intergenic spacer of 23 bp located between trnL1 and rrnL. All 13 PCGs used ATN as the start codon (cox1, atp6, and nad1 used ATA, nad2, nad3, nad5 and nad6 used ATT, cox2, cox3, nad4, nad4L, and cytb used ATG, atp8 used ATC), a total of 12 PCGs used TAA as the stop codon except ND3, which stopped with TAG.

To check the phylogenetic status of *M. mellinum*, we reconstructed a phylogenetic tree using all available mitogenome sequences of subfamily Syrphinae species in the NCBI database, and two Eristalinae species were used as an outgroup. Sequence alignments of 13 PCGs were generated by software MAFFT v7.313 with the E-INS-I strategy (Katoh and Standley 2013), and the best fit model of the partition scheme was determined by program PartitionFinder2 v2.1.1 with AICc scoring criteria (Lanfear et al. 2017). Phylogenetic trees were inferred with Bayesian inference (BI) and maximum likelihood (ML) by MrBayes v3.2.6 (Ronquist et al. 2012) and IQ-tree v1.6.8 (Nguyen et al. 2015), respectively. The tree topologies reconstructed by ML and BI methods were consistent (Figure 1) and supported the close relationships of *M. mellinum* with *M. orientale* within *Melanostoma* genus, but the monophyly of tribe Bacchini is not supported.

**Figure 1.**
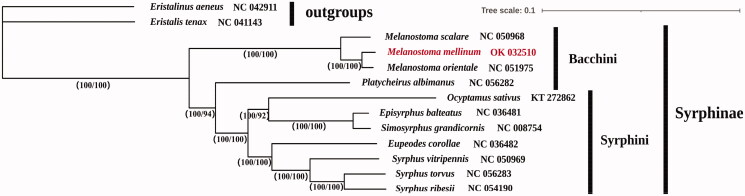
The Bayesian Inference (BI) and Maximum-Likelihood (ML) phylogenetic tree of Subfamily Syrphinae species based on the 13 PCGs. Statistical support values (Bootstrap/posterior probability) of ML/BI methods are shown below each node.

## Data Availability

The mitogenome sequence data that supported the findings in this study are openly available in GenBank of NCBI at (https://www.ncbi.nlm.nih.gov/) under the accession no. OK032510. The associated SRA, BioProject and BioSample numbers are SRR19141043, PRJNA836006 and SAMN28128889, respectively. The specimen was deposited at the Museum of Zoology and Botany, Shaanxi University of Technology, Hanzhong, China (https://www.snut.edu.cn/, Le Zhao, email: Lezhao@snut.edu.cn).
